# Concomitant Vitamin B1 and Vitamin B12 Deficiency Mimicking Thrombotic Thrombocytopenic Purpura

**DOI:** 10.7759/cureus.34421

**Published:** 2023-01-31

**Authors:** Venu M Ganipisetti, Pratyusha Bollimunta, Nyi Nyi Tun, Ashok Kanugula, Vishwanath Anil, Anand Athavale, Babu Sriram Maringanti

**Affiliations:** 1 Hospital Medicine, Presbyterian Hospital, Albuquerque, USA; 2 Hospital Medicine, Miami Valley Hospital, Dayton, USA; 3 Internal Medicine, Wellstar Spalding Regional Hospital, Griffin, USA; 4 Hospital Medicine, University of New Mexico, Albuquerque, USA

**Keywords:** mimic disease, vitamin b12 deficiency anemia, microangiopathic haemolytic anemia, hemolytic anaemia, neuro-psychiatric, vitamin b1 deficiency, wernicke encephalopathy, thrombotic thrombocytopenic purpura (ttp)-like syndrome, pseudo-ttp, vitamin b12 deficiency symptoms

## Abstract

Vitamin B12 deficiency-induced pseudo-thrombotic thrombocytopenic purpura (pseudo-TTP) is a rare condition. In reported literature, most cases were due to pernicious anemia (confirmed by the presence of anti-parietal cells or anti-intrinsic factor antibodies). Nutritional vitamin B12 deficiency causing pseudo-TTP is a much rarer entity. Differentiating thrombotic thrombocytopenic purpura (TTP) cases from pseudo-TTP (from any cause) should be done as soon as possible since the etiology, treatment, and outcome are different. Hematological findings from pseudo-TTP (when associated with vitamin B12 deficiency) respond to B12 replacement but do not respond to plasmapheresis. Neurological symptoms are one of the criteria for TTP, and altered mentation or psychosis in these cases is presumed secondary to either TTP or vitamin B12 deficiency. However, neurological symptoms are more characteristic of TTP rather than pseudo-TTP. In the rarer subsets of patients concerned with nutritional deficiency and neuropsychiatric symptoms, prompt consideration of concomitant vitamin B1 deficiency and Wernicke encephalopathy is essential. Immediate empiric treatment with high-dose IV thiamine should be started. If unrecognized and left untreated, thiamine deficiency can cause rapid progression to irreversible neurological symptoms, coma, and death, despite hematological improvement with B12 replacement. We report a rare case of concomitant vitamin B12 and vitamin B1 deficiency presenting with confusion, severe hemolytic anemia, acute renal failure, diarrhea, and thrombocytopenia mimicking TTP.

## Introduction

Pseudo-thrombotic thrombocytopenic purpura (pseudo-TTP) mimics the hematological presentation of TTP (a thrombotic microangiopathic syndrome), which comprises a pentad of hemolytic anemia, thrombocytopenia, fever, neurological symptoms, and renal failure but does not respond to TTP treatment [1]. Neurological symptoms and renal failure are not characteristic of pseudo-TTP [2]. Patients need emergent plasmapheresis in TTP cases, and pseudo-TTP requires identifying the cause and its treatment. Vitamin B12 deficiency has been reported to be an etiology causing hemolysis and thrombocytopenia mimicking TTP. Prompt recognition of the condition and treatment with parenteral B12 replacement is essential. Most reported vitamin B12 deficiency-induced pseudo-TTP cases in the literature are secondary to pernicious anemia, which is antibody-mediated disruption of B12 absorption [1,3,4]. We would like to highlight that in contrast to the majority of reported cases of vitamin B12- associated pseudo-TTP, which were from pernicious anemia, our patient had possible nutritional vitamin B12 deficiency along with vitamin B1 (thiamine) deficiency. If vitamin B12 deficiency is corrected alone, it will improve hematological abnormalities; however, without prompt and aggressive treatment of vitamin B1 deficiency, neurological symptoms may not improve and, in fact, may continue to worsen [5]. Neurological symptoms from vitamin B12 deficiency are more chronic and expected to show improvement starting from six weeks [6]. A confounding factor such as vitamin B1 deficiency complicating the picture can easily be missed, only to realize several weeks later that mentation has continued to decline rather than improve.

We report a case of an elderly patient with vitamin B12 deficiency presenting with TTP-like features, including severe hemolytic anemia, but had an overlap of acute neuropsychiatric symptoms from vitamin B1 deficiency. We started emergent plasmapheresis for presumed TTP; however, on further workup, it became more apparent that he had pseudo-TTP due to vitamin B12 deficiency with negative anti-intrinsic factor and anti-parietal antibodies. Curiously, his neuropsychiatric symptoms were not transient, unlike in other reported cases, and instead persisted. In the setting of neuropsychiatric symptoms with the underlying concern of malnutrition, we suspected concomitant vitamin B1 deficiency. We immediately started him on aggressive intravenous thiamine replacement, which improved his acute delirium in a few days. Later, low serum vitamin B1 levels confirmed the deficiency. If left untreated, vitamin B1 deficiency is known to cause irreversible neurological changes, including coma, and even cause death [5].

## Case presentation

An elderly American-Indian male in his 70s with no known past medical history, independent at baseline and living by himself, was brought to the emergency department for confusion, sleepiness, and generalized weakness. The patient could not provide reliable history due to altered mentation. Based on the limited history provided by the caregiver, who intermittently checks on him, the patient had been feeling increasingly weak with poor oral intake for several weeks, which acutely worsened over one week before the presentation. He had little appetite and was only having a few sips of broth, sodas, and coffee. They also reported a history of nausea, vomiting, and diarrhea. He had no history of heavy alcohol, nicotine, or other recreational drug use. The patient rarely follows up with health care providers, and there were no known past medical problems, prior surgeries, or recent hospitalizations. On exam, he was afebrile, his blood pressure was 60/40 mm Hg while sitting up with a heart rate of 105 beats per minute, respiratory rate was 14 per minute, and oxygen saturation was 90% on room air. His BMI was 18.9 kg/m^2^. He was confused and oriented to self only. He had dry mucus membranes. Heart and lung auscultation were normal. The abdomen was soft, non-tender, and non-distended. A neurological exam showed motor strength of four out of five all extremities; however, sensations, proprioception, and gait could not be assessed. Digital rectal exam and stool guaiac testing were negative.

Initial labs in the ER showed a low hemoglobin level, low platelet count, low haptoglobin level, high MCV value, high LDH, and increased bilirubin with normal AST, ALT, and alkaline phosphatase. He had increased creatinine, BUN levels, and normal serum electrolytes. His blood glucose level was normal. Initial sepsis screen was negative, including chest x-ray, covid screen, abdominal imaging, and urinalysis. Relevant initial labs are summarized in Table 1. Peripheral smear showed schistocytes, spherocytes, multiple teardrop cells, and hyper-segmented neutrophils. TTP was suspected due to the constellation of features, including hemolytic anemia, jaundice, severe thrombocytopenia, mental status changes, acute kidney injury, and diarrhea. The patient got transferred to our hospital for initiation of plasmapheresis. Before transfer, additional workup, including vitamin B12 level, was collected. The labs were sent out to an outside lab, and results were not readily available. He was given a dose of 1,000 mcg of intramuscular (IM) cyanocobalamin, solumedrol, fluid boluses, and three units of packed red blood cell transfusion.

**Table 1:** Lab values on initial presentation. WBC: White blood cell count; MCV: Mean corpuscular volume; RDW: Red cell distribution width; BUN: Blood urea nitrogen; LDH: Lactate dehydrogenase

Lab	Reported value	Reference Range
WBC	4.95 x 10E3/µL	3.5-11
Hemoglobin	3.2 g/dL	13.3-17.7
Hematocrit	10.4%	40-52
MCV	126.8 fL	80-100
RDW	38.4%	11.5-14.5
Platelet count	38 x 10E3/µL	150-400
Glucose	122 mg/dL	74-106
Creatinine	2.0 mg/dL	0.5-1.3
BUN	53 mg/dL	7-20
Total Bilirubin	6.9 mg/dL	0.6-1.3
Direct Bilirubin	1.9 mg/dL	<0.3
LDH	8107 U/L	100-190
Haptoglobin	<8 mg/dL	30-200
Reticulocyte index	1.1%	0.3-2.4

When the patient arrived at our facility, his BP was stable and renal function normalized with an improvement of hemoglobin to 7.1 gm/dL. However, he had persistent altered mental status. Additional labs were collected, including ADAMTS13. The patient was initiated on daily plasmapheresis. His calculated Plasmic score was 5 (Intermediate risk). No evidence of hepatosplenomegaly was noted on imaging. HIV, hepatitis screen, and DAT (Direct antiglobulin) were negative. Folate levels were normal. PT, PTT, INR, and fibrinogen levels were normal. D-dimer was elevated. MRI of the brain was negative for acute pathology, including stroke. CSF analysis was unremarkable and negative for infection, leukemic screen, and oligoclonal bands. The Syphilis screen was negative. Stool cultures could not be collected as the patient no longer had diarrhea after arrival at our center.

On day 4 of hospitalization, the ADAMTS13 level resulted at 28%, not consistent with TTP. A bone marrow biopsy was already done by this time due to a confusing picture, which showed megaloblastic changes compatible with healing vitamin B12 deficiency (likely from the one dose of empiric vitamin B12 injection he received at ER). He was still confused and agitated and intermittently required restraints. Records from the outside lab became available, which showed low vitamin B12 levels. Plasmapheresis and steroids were discontinued. We ordered serum vitamin B1 levels and started the patient on parenteral vitamin B12 1,000 mcg daily and intravenous thiamine 500 mg three times daily replacement. Subsequently, methylmalonic acid levels came high, along with increased homocysteine levels, confirming the diagnosis of vitamin B12 deficiency. Antiparietal cell antibodies and intrinsic factor-blocking antibodies were negative. Later, vitamin B1 levels came very low, supporting the diagnosis of concomitant vitamin B1 deficiency. We summarized the relevant labs done during the hospital course in Table 2.

**Table 2:** Additional important labs during the hospital course. Vitamin B12: cobalamin; vitamin B1: thiamine

Lab	Reported value	Reference range
Vitamin B12	64 pg/mL	193-986
Methylmalonic acid	3.85 µmol/L	<0.40
Homocysteine	22.5 µmol/L	3.2-10.7
Vitamin B1	<2 nmol/L	4-15
Folate	8.3 ng/mL	5.6-56.3

With vitamin B12 replacement therapy, platelet counts, and haptoglobin levels normalized by day 9, LDH was down-trending. There was a remarkable improvement in his mentation by day 12 (eight days after the initiation of vitamin B1 therapy). His hemoglobin levels remained stable. Daily lab trends are summarized in Table 3.

**Table 3:** Trend of lab values from day 1 to day 9 since the presentation. LDH: Lactate dehydrogenase

Lab (Reference range)	Day 1	Day 2	Day 3	Day 4	Day 5	Day 6	Day 7	Day 8	Day 9
Reticulocyte Index (0.3%-2.4%)		1.1		1.1	1.8	7.1	11.4	11.9	10.6
Hemoglobin (13.3-17.7 g/dL)	3.2	7.9	7.4	6.4	6.6	6.4	6.8	7.1	7.7
Platelets (150-400 10E3/µL)	38	28	25	14	13	19	47	100	152
LDH (100-190 U/L)	8107	2315	767	633	737	708	723	687	528
Haptoglobin (30-200 mg/dL)	<8	<8	36	<8	<8	<8	<8	12	37

After the patient became alert, he reported poor nutrition for several months due to financial constraints. We continued thiamine 500 mg IV three times daily for a week, followed by 250 mg IV daily for three more days before switching to 100 mg daily oral maintenance dose. Concomitantly, we treated him with daily parenteral vitamin B12 1,000 mcg for seven days and switched to weekly 1,000 mcg injections for four weeks to be followed with subsequent oral replacement.

Physical and occupational therapies evaluated him once he became more cooperative, and we discharged him to a skilled nursing facility in stable condition. He was referred to Gastroenterology for an upper GI endoscopy and colonoscopy to evaluate for any other gastrointestinal pathology that could contribute to nutritional deficiency and poor nutrition; however, patient elected not to pursue invasive procedures.

## Discussion

TTP is characterized by a pentad of fever, hemolytic anemia, thrombocytopenia, renal failure, and neurological symptoms. ADAMST13 inhibitor leads to increased Von Willebrand factor, which causes multimer formation, thrombosis, and end-organ damage. It is a hematological emergency, and treatment involves the emergent initiation of plasmapheresis, without which the condition can be fatal. On the other hand, Pseudo-TTP is defined as a condition presenting with features of TTP, such as labs suggestive of hemolysis and peripheral smear findings of schistocytes with or without thrombocytopenia but does not respond to the usual treatment of TTP (emergent plasmapheresis) [1]. Acute renal failure and neurological symptoms are relatively rare with Pseudo TTP, and these findings are more consistent with TTP [2] but do not essentially rule out the condition. Pseudo-TTP is treated by correcting the underlying condition, so accurate diagnosis and knowledge of this condition are essential. Differential diagnoses of pseudo-TTP with cytopenias include vitamin B12 deficiency, myelodysplastic syndrome, drug-induced TTP (including cocaine), and connective tissue disorders such as SLE [4]. With serological workup, drug screen, and bone marrow biopsy, we ruled out these alternate etiologies in our patient.

The true incidence of pseudo-TTP secondary to vitamin B12 deficiency is unknown due to the rarity of this condition and the paucity of published studies. A French study in 2006 based on 201 patients showed the incidence of pseudo-TTP in vitamin B12 deficient patients to be around 2.5% [7]. In contrast, a more recent retrospective cohort study by Koshy et al. on 2,669 patients with vitamin B12 deficiency reported that the prevalence could be as low as 0.6% [3]. The majority of the pseudo-TTP in vitamin B12 deficit in the previously reported studies and case reports was secondary to pernicious anemia (65%-70%), and secondary to nutritional deficiency was a small subset [1,3,4]. Hence in the majority of cases (65%-70% with Pernicious anemia), treatment with B12 would suffice. However, in minority patients with nutritional deficiency and neuropsychiatric symptoms, exceptional attention to vitamin B1 deficiency should be given.

Vitamin B1 deficiency can develop in four to six weeks of poor nutrition and cause Wernicke encephalopathy, which is characterized by a triad of Ophthalmoplegia, gait ataxia, and delirium; however, all three are present only in 17% of cases and delirium is the most common symptom on presentation [5]. When suspected, prompt empiric treatment without delay is critical as without this, Wernicke encephalopathy can lead to permanent brain damage, coma, or death [5].

Our patient presented with laboratory features of TTP, neuropsychiatric symptoms, and concern about poor nutrition. Upon a literature review of existing vitamin B12 deficiency-associated pseudo-TTP case reports, severe mental status changes were rarely reported. However, transient mentation change in two reported cases was attributed to hypovolemia and anemia, evident from rapid improvement with resuscitation [8,9]. Our patient was initially hypotensive with findings of severe anemia and was given adequate fluid resuscitation and transfusions. Despite this, neuropsychiatric symptoms persisted. Concomitant vitamin B1 deficiency was suspected, and he was empirically started on high-dose IV thiamine along with B12 replacement. After vitamin B12 deficiency treatment, improvement in neuropsychiatric symptoms may take longer, starting in about six weeks to three months and continuing to improve for up to a year [6]. In contrast, prompt treatment of Wernicke encephalopathy improves confusion in these patients within days to weeks [10]. Our patient’s mentation started improving within a week of starting high-dose thiamine replacement, suggesting the role of vitamin B1 deficiency to be a significant contributor to his neuropsychiatric symptoms. Later, his vitamin B1 levels also came low, supporting the diagnosis further.

Authors of previously published case reports attempted to develop a specific diagnostic criterion for Pseudo TTP with vitamin B12 deficiency. Some characteristics included a high LDH, high bilirubin, hypersegmented neutrophils, low reticulocyte index, low to medium plasmic score, schistocytes, and teardrop cells on peripheral smear, etc. [3,4]. Our patient’s labs were in alignment with these diagnostic criteria.

## Conclusions

It is essential to recognize pseudo-TTP, identify the underlying etiology for prompt treatment, and avoid unnecessary plasmapheresis. The case also highlights the importance of acknowledging that neuropsychiatric symptoms are not typically associated with pseudo-TTP. Treatment with B12 replacement can resolve hematological abnormalities immediately in nutritionally deficient patients, giving false reassurance about improvement. However, irreversible neurological symptoms/damage can occur when concomitant vitamin B1 deficiency is not suspected and treated promptly.
